# Incorporating Community Partner Perspectives on eHealth Technology Data Sharing Practices for the California Early Psychosis Intervention Network: Qualitative Focus Group Study With a User-Centered Design Approach

**DOI:** 10.2196/44194

**Published:** 2023-11-14

**Authors:** Laura M Tully, Kathleen E Nye, Sabrina Ereshefsky, Valerie L Tryon, Christopher Komei Hakusui, Mark Savill, Tara A Niendam

**Affiliations:** 1 Department of Psychiatry and Behavioral Sciences University of California, Davis Sacramento, CA United States; 2 Department of Psychiatry University of California, San Francisco San Francisco, CA United States

**Keywords:** attitude, content analysis, data sharing, eHealth, ethic, focus group, health information exchange, mental health, perspective, preference, psychosis, psychotic, qualitative data

## Abstract

**Background:**

Increased use of eHealth technology and user data to drive early identification and intervention algorithms in early psychosis (EP) necessitates the implementation of ethical data use practices to increase user acceptability and trust.

**Objective:**

First, the study explored EP community partner perspectives on data sharing best practices, including beliefs, attitudes, and preferences for ethical data sharing and how best to present end-user license agreements (EULAs). Second, we present a test case of adopting a user-centered design approach to develop a EULA protocol consistent with community partner perspectives and priorities.

**Methods:**

We conducted an exploratory, qualitative, and focus group–based study exploring mental health data sharing and privacy preferences among individuals involved in delivering or receiving EP care within the California Early Psychosis Intervention Network. Key themes were identified through a content analysis of focus group transcripts. Additionally, we conducted workshops using a user-centered design approach to develop a EULA that addresses participant priorities.

**Results:**

In total, 24 participants took part in the study (14 EP providers, 6 clients, and 4 family members). Participants reported being receptive to data sharing despite being acutely aware of widespread third-party sharing across digital domains, the risk of breaches, and motives hidden in the legal language of EULAs. Consequently, they reported feeling a loss of control and a lack of protection over their data. Participants indicated these concerns could be mitigated through user-level control for data sharing with third parties and an understandable, transparent EULA, including multiple presentation modalities, text at no more than an eighth-grade reading level, and a clear definition of key terms. These findings were successfully integrated into the development of a EULA and data opt-in process that resulted in 88.1% (421/478) of clients who reviewed the video agreeing to share data.

**Conclusions:**

Many of the factors considered pertinent to informing data sharing practices in a mental health setting are consistent among clients, family members, and providers delivering or receiving EP care. These community partners’ priorities can be successfully incorporated into developing EULA practices that can lead to high voluntary data sharing rates.

## Introduction

The past decade has seen a rapid expansion in the availability of eHealth technology (eg, smartphone and tablet applications and web-based portals) to support individuals with psychosis [1]. Individuals with psychosis are willing and interested in using eHealth technology as part of their care [2-5]. eHealth tools promote treatment engagement [6], symptom monitoring [7,8], relapse prediction [9] and enhance quality of life [10] and functioning [11]. Consequently, industry developers and academics are racing to implement eHealth technology at scale to improve outcomes for those experiencing serious mental illness.

As eHealth technology advances and we leverage user data to drive early identification and intervention algorithms [12], it is imperative that we implement ethical data use standards. Typical software has long end-user license agreements (EULAs) replete with legal jargon detailing the myriad ways user data are used and shared [13] with little or no user control. Therefore, users frequently report that they rarely read the EULA and may not understand what they are agreeing to [14,15]. Such concerns have led some to question whether the EULA should be considered an effective tool for informed consent, with concerns that the agreement typically serves to protect the company but not the user [16]. As a result, technology users may unknowingly have their data shared or sold to third parties, sometimes without encryption, rendering data vulnerable to privacy breaches [13,17-21]. These issues may be particularly relevant in psychosis, given that cognitive impairments associated with psychotic disorder could impact EULA comprehension—data breaches of sensitive and highly stigmatized psychosis diagnoses could be especially harmful.

Users have varied attitudes about risk: some report skepticism of eHealth data [13,16]; others feel cognitive dissonance around risks as a reality of using digital platforms, especially those that are “free” in return for data use [19,22,23]. However, health data are personal and private—researchers, providers, and industry partners alike have a duty to protect vulnerable individuals from data misuse. Moreover, an outcomes-driven health care system (an agreed goal in the health care industry [24]) relies on large, interagency data sharing. To do this, we must implement ethical data use practices to increase user acceptability and trust in eHealth platforms.

One such effort to build an outcomes-driven health care system is the California Early Psychosis Intervention Network (EPI-CAL). EPI-CAL is a multiyear project that connects early psychosis (EP) programs across California through an eHealth application, Beehive, in a learning health care network [25]. Beehive facilitates client-, family-, and clinic-level outcomes data collection as part of regular care across EP programs using a battery of validated measures. Adopting a learning health care network approach to psychosis care has the potential to support innovation, improve efficiency, and improve care delivery and outcomes [26]. EPI-CAL’s design relies on clients with EP “choosing” to share their data for analysis outside of standard clinical care by agreeing to a EULA that allows the software to be used to collect, transfer, and present client data. To create an adequate EULA in this setting, previous research suggests that EULAs should be relevant and understandable [27], use video explanations [28,29], set the reading level to sixth to eighth grade [27,30], include comprehension checks [31,32], offer explicit “opt-in” selections [16,30,33,34], and include options to request ending data collection or delete data entirely [30]. Unfortunately, such proposals are rarely implemented in practice [35], and therefore, our team sought to elicit feedback from relevant community partners to inform the design of a EULA that incorporates best practices for informed data sharing in an EP setting.

In the first phase of the study, the aim was to explore family members, clients, and EP care providers’ beliefs, attitudes, and perspectives on ethical data sharing in EP settings. These findings were then used to develop a EULA for our eHealth data collection platform, appropriate for use in an EP treatment setting. In the second phase, we presented our EULA materials to family members, clients, and EP care providers with the aim of understanding (1) to what extent these materials addressed their concerns and priorities and (2) what features could be amended to better meet the goal of developing an accessible, transparent, and flexible EULA. Therefore, the first phase serves to explore generalizable principles of ethical data sharing practices relevant to an EP setting. The second phase represents a case example of using a user-centered design approach to developing eHealth data sharing practices [10,36,37], informed by the perspectives of participants provided during phase 1.

## Methods

### Design

We used a two-phase approach: (1) an exploratory, qualitative, and focus group–based study design to explore participants’ mental health data sharing and (2) a privacy preferences and a user-centered design workshop design to evaluate implementation of the perspectives shared by participants in the first phase of the study. We used the COREQ (Consolidated Criteria for Reporting Qualitative Research) checklist to guide the design and implementation of the study [38] (Multimedia Appendix 1).

### Recruitment

We recruited participants from three EP community partner groups: (1) clinical staff and providers, (2) clients, and (3) family members of clients. Eligible participants were (1) actively or formerly affiliated with an EPI-CAL EP clinic, (2) English-speaking, and (3) able to provide written informed consent and assent (minors).

EP provider participants were recruited through research team contact with the team lead of the 12 active EPI-CAL EP programs, asking if at least 1 provider or staff could participate. We used this approach to ensure a maximal number of EPI-CAL programs were represented and to minimize overrepresentation from a small number of clinics. Client and family participants were invited either through clinician referral or by the research team directly contacting individuals who had previously given permission to be contacted for future research opportunities.

### Data Collection and Analysis

The development of the phase 1 focus group interview guide was grounded in (1) the authors’ previous clinical and research experience implementing eHealth in EP care [7,8], (2) the authors’ own questions regarding how to best inform individuals about how their data would be used in clinical care and research as part of the impending implementation of Beehive within EPI-CAL, and (3) a brief review of the relevant literature [16,21,39]. The developed focus group guide extends the work of Shen et al [21], who created an interview guide to assess the privacy and data sharing experiences and perspectives of individuals with mood, anxiety, and substance use issues. Additionally, our guide incorporates ideas from Stopczynski [39], who suggested that best practice should emphasize the end user over the research, allowing the “end user” to feel empowered to exercise control over their data. Some specific user-centered design elements include having data sharing access options, having the ability to change one’s mind, using simple language, and understanding content through multimedia inputs. Finally, the work of Torous et al [16] was incorporated, which recommends the involvement of community partners from the beginning of any eHealth application development, ensuring the inclusion of EULA comprehension checks and including explicit agreement sharing options.

The phase 1 focus group guide (Multimedia Appendix 2) began with defining key concepts relevant to sharing and using health information collected through an eHealth platform, including privacy, confidentiality, and the distinction between deidentified and anonymous information. The remaining questions prompted participants to share their understanding and perspectives on (1) data sharing, (2) changing sharing options, and (3) sharing different types of data (eg, identifiable vs deidentified) at different levels (eg, individual- and group-levels). Descriptive ice-breaker questions (Multimedia Appendix 3) were administered as a poll at points throughout the group to generate discussion, allow private reflection, and increase engagement.

During phase 1, we conducted three 90-minute focus groups, including 1 client, 1 family member, and 1 provider group. These focus groups were conducted during August 2020 through videoconferencing to comply with COVID-19 restrictions at the time. Each group included a facilitator (LMT or SE), cofacilitator (SE or KEN), and note taker (KEN or CKH). There were no other individuals present other than researchers and participants. The positionality of each researcher is detailed in Table 1. Each group began with the introduction of the research team, including their occupation and the role they would have in the focus group. After each group, the research team met to discuss any salient points and preliminary themes. These reflections were used to refine the focus group guide before conducting a subsequent group.

**Table 1:** Positionality of the research team that conducted groups and analyzed the qualitative transcripts.

Researcher initials	Credentials	Occupation	Gender	Experience and training
LMT	PhD (clinical psychology)	Academic researcher and clinical psychologist	Nonbinary	Licensed clinical psychologist with expertise in early psychosis
KEN	BA	Academic researcher	Female	Clinical and research experience working with individuals with early psychosis and training in qualitative data collection and coding
SE	PhD (clinical psychology)	Academic researcher and clinical psychologist	Female	Postdoctoral research scholar with expertise in early psychosis and training in qualitative data collection and coding
VLT	PhD (behavioral neuroscience)	Academic researcher	Female	Training in qualitative data collection and coding
CKH	BA	Academic researcher	Male	Lived experience navigating the US mental health system

Each group was audio recorded. Upon the completion of each phase, these recordings were transcribed, cleaned, and hand-coded using directed content analysis [40]. In this approach, the coding team (KEN, SE, VLT, and LMT) first reviewed the transcripts, highlighting identified ethical data sharing themes. Next, the coding team developed a preliminary coding framework based on the examined text, informed by preexisting literature concerning ethical behavioral health data sharing principles [16,21,39]. Next, 2 authors (KEN and SE) independently coded each transcript using the developed coding framework, compared their responses, and resolved any disagreements through discussion. Where appropriate, this coding framework was iteratively revised as new codes emerged. From these codes, a set of categories was developed, and then major and minor themes were established. All analysis was conducted using NVivo qualitative analysis software (QSR International).

In phase 2, using the findings from the phase 1 focus group, the research team created an informational whiteboard Beehive EULA video (Multimedia Appendix 4) explaining data sharing in the application, the choices that each user would have to share their data for research, and a visualized Beehive data sharing screen, which presented opt-in choices of data sharing levels to users after watching the EULA video. Next, the guide for the phase 2 workshop (Multimedia Appendix 5) was developed; it focused on reviewing the developed materials and eliciting feedback on the approach, the user interface, and the information presented. In the workshops, all participants watched the EULA video twice before reviewing the opt-in data sharing screen.

The phase 2 workshop transcripts were coded by 2 authors (KEN and VLT) and analyzed using an approach consistent with the phase 1 focus groups. Once the research team completed a preliminary draft of the coding framework, participants were contacted 1 final time and emailed the major and minor themes, supported by key quotations, from their research participation activities. Participants could provide feedback through a survey (Multimedia Appendix 6) or through videoconference discussion with researchers (KEN, SE, and VLT). This feedback then informed the structure of the coding framework. Once analysis was completed, based on the data, a series of modifications were made to both the EULA video and the user interface for the data sharing screen.

### Ethical Considerations

The institutional review board of the University of California, Davis, approved the study (1403828-21, California Collaborative Network to Promote Data-Driven Care and Improve Outcomes in Early Psychosis [CORE]). Additionally, several of the EP program participating counties and universities in EPI-CAL required a separate review of the project by their institutional review board, which provided their approval. All study participants provided written informed consent and assent (as appropriate). Participants received US $30 compensation for each focus group (they could participate in both).

## Results

### Participants

At least 1 provider participant from 12 EPI-CAL programs participated in the study. The clinical roles of these participants included clinicians, case managers, supported employment and education specialists, clinic coordinators, clinical supervisors, and program directors. These roles are not specified with quotations in order to protect the identities of participants.

Regarding client and family recruitment, 30 individuals were contacted directly by the research team. An unknown number of clients and family members were introduced to the study by their respective providers in the 12 EPI-CAL programs. Of all the clients and family members introduced to the study, 10 (6 clients and 4 family members) agreed to participate. Of the 20 who were directly contacted by the research team and did not participate, most (n=12, 60%) did not respond to recruitment attempts; a few (n=3, 15%) stated they were not available; and 5, who initially agreed to participate, ultimately did not attend the research activity. Therefore, the final sample included 24 participants (14 providers, 6 clients, and 4 family members). Participant demographics are presented in Table 2.

Following the completion of the preliminary coding framework, attempts to contact all participants were made, and 8 participants in total (3 clients, 4 providers, and 1 family member) agreed to provide feedback: 6 through a survey and 2 through a videoconference. Overall, participants agreed with the identified themes, and as a result, no significant changes were made to the coding frameworks. Some researchers had existing professional relationships with some participants due to previous research or contact at EPI-CAL focus groups.

**Table 2:** Demographic and clinical characteristics of participants.

Characteristics			All (n=24)		EP^a^ providers and staff (n=14)		EP clients (n=6)		EP family and support persons (n=4)
Age (years), mean (SD; range)			36 (11.07; 16-56)		37.93 (9.02; 24-56)		23.83 (3.93; 16-28)		47.50 (7.76; 39-59)
Male sex, n (%)			9 (38)		4 (29)		4 (67)		1 (25)
**Race^b^, n (%)**									
	African American or Black	7 (29)		2 (14)		3 (50)		2 (50)	
	Asian	5 (21)		2 (14)		2 (33)		1 (25)	
	Hispanic ethnicity	6 (25)		5 (36)		1 (17)		0 (0)	
	Native American	2 (8)		1 (7)		1 (17)		0 (0)	
	Pacific Islander	1 (4)		1 (7)		0 (0)		0 (0)	
	White	13 (54)		9 (64)		2 (33)		2 (50)	
	Other	4 (17)		4 (29)		0 (0)		0 (0)	
**Gender identity, n (%)**									
	Male	6 (25)		4 (29)		1 (17)		1 (25)	
	Female	15 (63)		10 (71)		2 (33)		3 (75)	
	Nonbinary	3 (13)		0 (0)		3 (50)		0 (0)	
**Sexual orientation at baseline^c,d^, n (%)**									
	Heterosexual	19 (79)		13 (93)		2 (33)		4 (100)	
	Pansexual	1 (4)		0 (0)		1 (17)		0 (0)	
	No response	1 (4)		1 (7)		0 (0)		0 (0)	
	Other	3 (13)		0 (0)		3 (50)		0 (0)	

^a^EP: early psychosis.

^b^Participants can select more than 1 race; therefore, percentages might not sum to 100.

^c^Some participants changed their responses to this question between group 1 and group 2.

^d^Possible responses for sexual orientation that were not endorsed by any participants were “gay or lesbian,” “bisexual,” and “asexual.”

### Phase 1: Participants Attitudes and Understanding of Health Data Sharing

#### Overview

In the phase 1 focus groups, participants started by providing their perspectives on sharing their mental health data and factors that would affect their comfort with sharing. Overall, clients and family members reported feeling comfortable with sharing mental health data in a clinical setting. While we presumed mental health data to be more sensitive and thus have distinct considerations for sharing, many participants considered mental health data equivalent to physical health data; instead, they were more concerned with sharing personal information overall. Indeed, participants appeared to be very mindful of potential risks concerning data sharing.

I don’t have any distinction. I’m very open about my mental health as well as my physical.

Client 3, group 2

I feel like no data is safe. Once you release it onto the internet especially because of all the articles saying that there was a breach with this site, and they have your credit card information.

Provider 1, group 1

Participants indicated that multiple factors informed their decision-making process with regard to mental health data sharing. While some were specific as to what could be addressed by a EULA, it was notable that many other considerations that were nonspecific to the EULA process were also highlighted. A summary of these EULA-specific and more general factors is discussed below. Additional quotes supporting the main themes are presented in Multimedia Appendix 7.

#### EULA-Relevant Factors That Inform Decision-Making Regarding Mental Health Data Sharing

##### Overview

Factors that informed decision-making regarding data sharing that could be specifically addressed by a EULA and subsequent data sharing practices corresponded to four broad themes: (1) the importance of the EULA providing the necessary information required to make an informed decision and transparency around when and how the data will be used; (2) the degree to which clients have control and agency over the data they provide; (3) the degree to which appropriate data security practices are implemented and an explanation of how security would be maintained; and (4) clearly defined benefits derived from the sharing of personal data. A summary of each theme is presented below.

##### Transparency and Provision of Relevant Information

Transparency was considered foundational in participants’ data sharing calculus—paramount to this was knowing what, when, with whom, how, and why data are shared, including the disclosure of conflicts of interest and using layperson’s and culturally appropriate terms. The opportunity to review research results was 1 example of transparency that improved participants’ understanding of how data are used. Clinic participants suggested explaining current data protection laws may increase willingness to share data.

I just feel like I should be able to know who’s accessing what, when, and why. You know?

Parent 1, group 3

I need to know what is the formula [to deidentify data] like. You’ve described it to me, but that doesn’t give me the confidence to really give you a thumbs up.

Parent 4, group 3

##### Control and Agency of Data

Participants emphasized the importance of having control over their data, including sharing the minimum data necessary, restricting access, having access to the data themselves, having the ability to change one’s mind to facilitate no regrets (including being able to opt-in later)*,* and deleting data to give peace of mind. All participants noted that the limitations of deleting deidentified data should be clear, especially if data have been shared with outside parties.

I think [the ability to delete your data] is a fairly important option. If at the very least for the peace of mind it can give.

Client 3, group 2

There’s so many protections on my information that even I can’t access it, which I find really ridiculous... Why would I want you to share that information to other people if you won’t even share it to me?

Client 4, group 2

##### Data Security and Protections

Individuals want to know that the institution or entity to which they are entrusting their data is competent in upholding legal protections and that their information is protected and not sold to third parties. Clients emphasized that extra protections should be in place when individuals are in a vulnerable state (eg, a mental health crisis). Participants noted that clarity on the data only being presented in the aggregate was also important. It was notable that clients and family members were aware of at least some of the existing laws concerning data sharing, including that the Health Insurance Portability and Accountability Act (HIPAA) protects against the improper sharing of medical information.

I think if you’re not being identified I’m always willing to share a little bit more as we’re not going to be individualized.

Parent 3, group 3

Anytime data needs to be shared, I have to sign a paper to give permission.

Parent 1, group 3

##### Clarity Regarding Potential Benefits of Data Sharing

Clients, providers, and family participants all highlighted that a clear explanation of the benefits of data collection is an important consideration in agreeing to share data. Some focused on the personal benefits of data collection, such as supporting continuity of care or having data integrated into care delivery. However, others also highlighted the value of knowing how the data can support program sustainability and advance the field of EP care more broadly. This concept highlights a need for those collecting data to clearly define the benefits for users—for those who are providing their data—and those benefits should be clearly communicated or accessible before using that data.

If my therapist was going on a vacation leave, and then a new therapist was taking over, I think some basic information I’d at least want them to know, is my name, my age, I’m working or going to school, who I live with. If I hang out with friends, what my formal diagnosis is. I think these are all important things.

Client 4, group 2

I’m more comfortable sharing my information knowing that it’s going towards helping other people. And also funding too because I know that’s definitely important with further helping others as well.

Client 2, group 2

#### Factors Distinct From the EULA That Inform Decision-Making Regarding Mental Health Data Sharing

##### Previous Data Sharing Experiences

Previous experience, both positive and negative, influenced understanding and willingness to share data. Participants’ past experiences of data being held securely and appropriately increased comfort in sharing data in the future. Conversely, experiences where data were shared without their knowledge or ability to control it resulted in individuals feeling less comfortable about data sharing in the future. This underscores the importance of integrity in the use of data and how unethical practices can lead to a diminished willingness to share data in the future.

My son got dinged by the DMV due to hospital stay. Why would you do that if his record is way cleaner than mine driving-wise? There was no reason for him to get that letter in the mail saying you’re going to be suspended if you don’t show up at this court hearing. And that’s how it was derived: from the hospital stay.

Parent 3, group 3

I think my positive and negative biases are related to the fact that I’ve worked in clinical research for 20 years. And I wrote “somewhat comfortable” on both answers, because I know at our clinic, we’re super careful about how we collect [data].

Provider 2, group 1

##### Rapport Developed With Clinical Program

When researchers cannot be in direct contact with participants, they rely on established rapport between client and clinic staff, as staff are often the individuals who relay information about research opportunities. One clinician stated that “understanding what the purpose of the research is and how it’s helpful” can be a conduit for transparency. A clinical research coordinator noted that rapport alone is insufficient; clinicians must be able to explain the study.

I think rapport with our patients is really important... I think there was something about the rapport building up front from the phone line to actually consenting that was much more comfortable compared to just someone new coming in and explaining the consent that they had never had contact with or any relationship with prior.

Provider 3, group 1

### Phase 2: Developing a EULA Informed by Community Partner Perspectives on Ethical Mental Health Data Sharing

#### Development of EULA Materials

After completing the phase 1 focus groups, we (1) developed a whiteboard-style informational EULA video and (2) designed the user interface in Beehive on which users review the text of the EULA and make decisions about how they want their data to be used. This happened concurrently with the coding of phase 1 groups, with themes from these groups informing the development of these EULA materials.

While it was notable that multiple factors distinct from the EULA were considered important to decision-making regarding data sharing, issues concerning transparency, data protection and security, potential benefits, and control were considered important and something that could be specifically addressed by a EULA. In response to these findings, our informational video and text EULA were designed to include information in plain language regarding the purpose of data collection, the funders sponsoring the project, the entities who would have access to data and at what levels (identified vs deidentified), and how their data were secured and stored. We also provided information about how their participation in this project and sharing their data could benefit them and the population with EP in California more generally. Both formats of the EULA included information regarding opting into and changing data sharing permissions (ie, “control”). A detailed summary of how these themes were incorporated into the development of the EULA materials is presented in Table 3.

**Table 3:** Implementation of phase 1 themes into the Beehive end-user license agreement (EULA) video.

Theme	Transparency	Data protections	Control	Explanation of potential benefits
EULA text and video script	Eighth-grade reading level Explains what kinds of data are shared, who they are shared with, and why they are shared Will be translated into 12 additional threshold languages to serve the diverse population represented in EPI-CAL^a^ sites	Described in the context of both clinical care and protections for research data	Opt-in (vs opt-out) to data sharing with research Makes clear that sharing data with research is optional and using Beehive is not required to receive care Describes that users can change their mind	Added text toward the end of the video to explicitly describe the potential benefits of each level of data sharing
Application user interface	Bold text for each main point, with important subtext beneath each point	—^b^	User can submit to the EULA (and use application) without agreeing to data sharing for research Separate check boxes for each type of sharing Opt-in (vs opt-out) to share data with research	—
Video design	Important phrases and words written out These phrases and words will be translated into 12 additional threshold languages Graphics showing the relationship between entities	Give a clear visualization of how data are deidentified	Provide a clear visualization of the individual requesting to delete data	Provided a clear visualization of the text that describes the benefits of data sharing

^a^EPI-CAL: California Early Psychosis Intervention Network.

^b^Not addressed in the user interface.

#### Participant Perspectives on How the EULA Addresses Issues Related to Transparency, Control, Data Protection, and Potential Benefits of Data Sharing

Following the preliminary development of the EULA materials, we conducted user-centered workshops with the aim of soliciting feedback on the materials and focusing on potential areas for improvement. During these workshops, we presented Beehive EULA materials to participants through a whiteboard video and the application’s user interface, where users could indicate their data sharing choices.

Overall, the feedback from the participants was positive. Most considered the EULA to be highly transparent, although some clinicians were concerned with the relevance of particular visualizations, while a client participant suggested the term “deletion” of data may be misleading in this context. Others appreciated how the EULA provided agency and control back to the client, which is particularly important in this setting, given that individuals with psychosis can frequently feel that their agency is being taken away. Others reported that a key takeaway message from the EULA video was that they felt their data were secure, which was considered an important factor in agreeing to data sharing. Finally, feedback regarding the benefits of data collection was somewhat mixed. Some participants appreciated the fact that the EULA made clear how these data linked to the larger EPI-CAL research project centered on improving and evaluating outcomes. On the other hand, others were less clear on how data collection may lead to localized benefits, which raised concerns about the utility of the data being requested.

They feel like they don’t have a lot of self-control over things, or even their life, and this gives them control over at least this portion of it. And asking the questions beforehand to get permission before you put in any data, I think is an awesome idea.

Parent 2, group 6

[The message I came away with was] That my health information would be protected.

Provider 4, group 4

**Figure 1:**
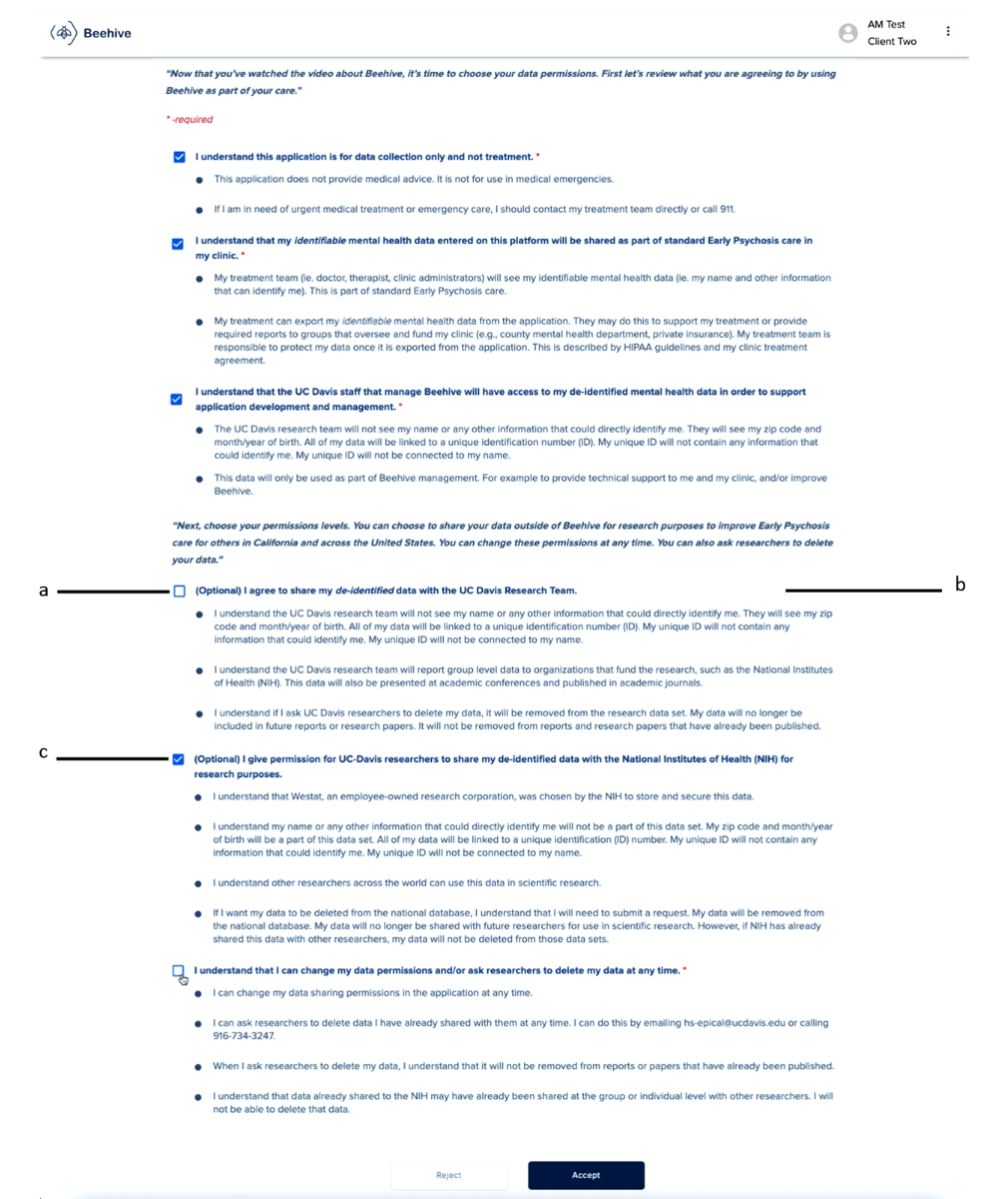
The Beehive end-user license agreement screen as presented in phase 2 focus groups was designed with feedback from phase 1 focus groups. Item “a” was from client input (phase 2—impact on transparency), item “b” was from client input (phase 2—impact on transparency), and item “c” was from support person input (phase 2—impact on transparency).

**Figure 2:**
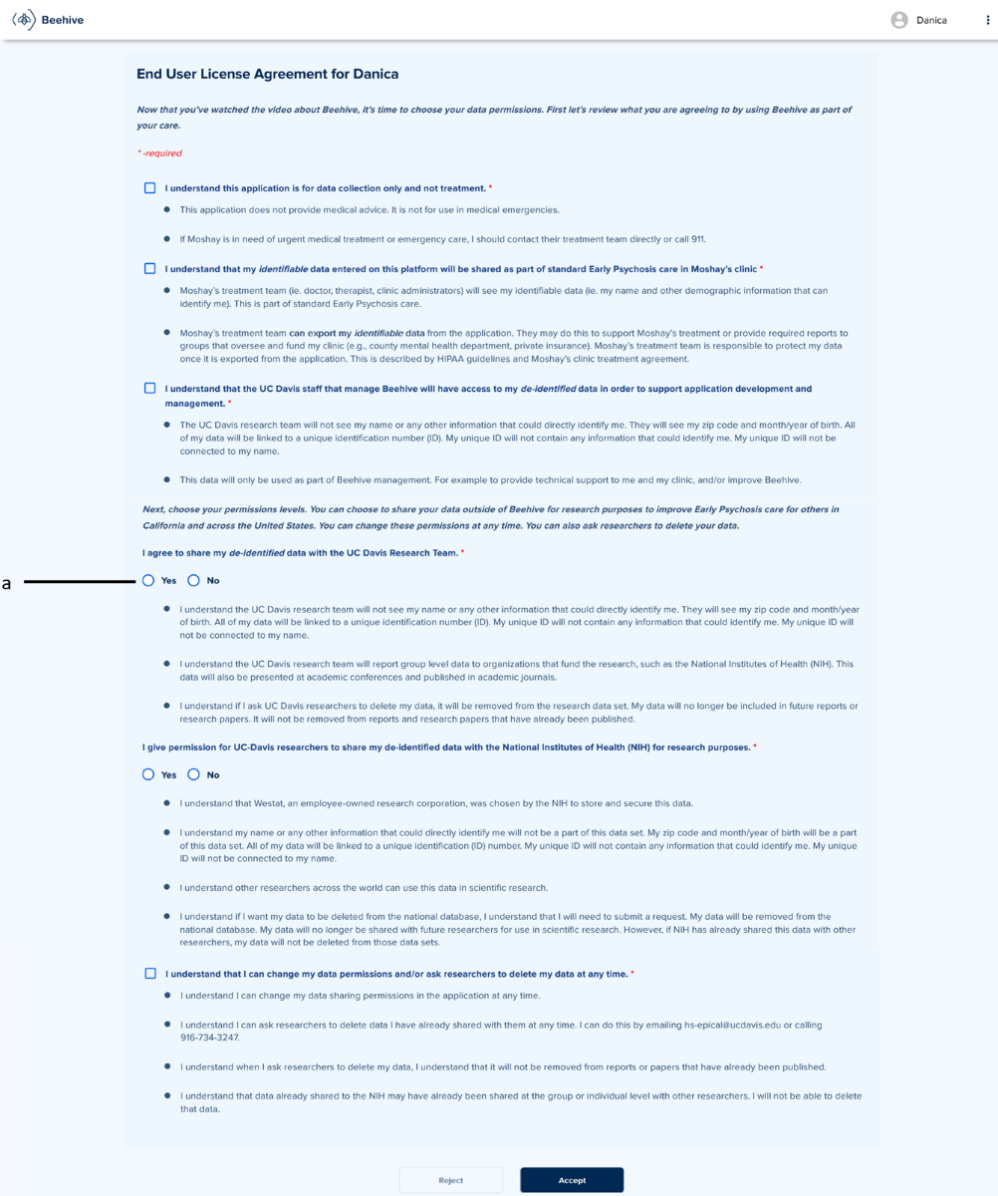
The Beehive end-user license agreement screen was updated based on feedback from phase 2 focus groups. Item “a” was from early psychosis team input (phase 2—impact on transparency).

Based on the feedback from participants during the phase 2 workshops, a series of modifications were made to the EULA. Examples include clarifying the research team’s access to deidentified data for quality management purposes, highlighting potential benefits to clients, further simplifying the text, and slowing the rate of speech. Additionally, we updated the user interface by changing the “opt-in” data sharing choices to a forced response (yes or no) regarding data sharing (Figures 1 and 2).

### Implementation of the Co-Designed EULA in EPI-CAL

The co-designed EULA was integrated into the EPI-CAL when the beta version of Beehive was launched on March 15, 2021. As of May 26, 2023, 475 clients have reviewed the EULA. Of these, 87% (n=412) of users have chosen to share their data with University of California, Davis researchers, and 83% (n=393) have chosen to share their data with the National Institutes of Health. Only a minority of clients (n=3, 0.6%) have withdrawn their permission to share data after initially choosing to share it.

## Discussion

This study explored EP community partner perspectives on ethical data sharing practices and what impacted their willingness to share data on eHealth platforms. Then, using these data, we developed a user-centered, accessible, transparent, and flexible EULA that aimed to incorporate EP community partner priorities. In the second phase of this study, we piloted the newly developed EULA materials with EP community partners in a user-centered design workshop format to evaluate if our EULA approach addressed the most critical elements needed for ethical data sharing practices. Community partners expressed overall positive attitudes toward the EULA materials and reported that the EULA would likely increase EP program participants willingness to engage in data sharing if they were using Beehive. This theoretical engagement with Beehive mentioned by participants is supported in practice by the high proportion of clients that have agreed to share their data after reviewing the Beehive EULA as part of their regular care. These findings, therefore, present 1 possible ethical framework for eHealth platforms adopting user-centered approaches. eHealth platforms developed with ethical data sharing practices can address client and family member priorities, which can also lead to a high proportion of clients with EP agreeing to share data.

In the focus group phase of the study, we elicited feedback from participants around sharing and using health information collected through an eHealth platform. We found major themes centered on data sharing practices that could be addressed by a well-designed EULA, as well as factors that were related to data sharing practices more generally. Regarding EULA relevant factors that would increase willingness to share data, four main findings emerged: focus group participants endorsed the core themes of (1) transparency, (2) data protections and limitations, (3) control and agency over the use of their data, and (4) clarity around the potential benefits of data sharing. Factors that influenced decisions around data sharing that could not be addressed by a EULA included past experiences with data sharing and rapport developed with clinical service providers facilitating data collection activities. These findings build on previous research highlighting a range of privacy-adjacent concerns [22,27,29,33], including transparency [27,41], relevancy [27], user-level control [41,42], and comprehension [13,20,43]. This demonstrates users’ desire to know the “what,” “when,” “how,” “why,” and “with whom” to make informed data sharing decisions. eHealth platforms need to equip users with enough information in their EULAs to objectively assess the benefits and risks of sharing their sensitive personal information.

EULAs typically have low readership [44], and profit-oriented applications aim to collect massive amounts of data [22]. Thus, there are minimal, if any, safeguards in place for vulnerable individuals. Even when deidentified data are used, they are often exempt from regulatory review [45]. As such, the EULA does not parallel the clinical or research-informed consent framework, and there is much that can be applied regarding the ethical use of eHealth technology. Though informed consent is required to cover aspects of privacy, risks, and ethical use of data, it still falls short in similar ways to the typical EULA, such as falling into the trap of long, technical, and difficult-to-understand language (ie, above recommended reading levels), and often requiring supplemental scripts describing the process in more granular steps, using plain layperson’s terms, and requiring comprehension checks [20,28,31,32,34,46-48], though this has historically not been a standardized process [49]. The goal of this project was to respond to previous EULA and consent framework limitations and address the concerns that users had. These closely aligned with the themes of transparency and comprehension, protections, control, and explanation of potential benefits observed in our focus groups.

Our results demonstrate the value of partnering with community members to develop eHealth technology and related EULA materials. Participants’ wide range of experiences and perspectives emphasized their desire for control and protection over their data. Workshop participants upheld the importance of allowing users to change their data sharing preferences at any time; they viewed such a feature as a way to support vulnerable individuals who may wish to modify data sharing decisions they made during times of sedation from psychotropic medications, for example. Similarly, participants highlighted the impact of trust and rapport between client and provider on data sharing decisions; they suggested that providers review the EULA video with clients and families to answer questions and provide encouragement and assurance as they consider their data sharing options. This indicates that person-to-person discussion of the EULA also impacts comprehension, comfort using eHealth technology, and whether the user chooses to share their data. By centering the voices of users, we gained valuable insight into how best to balance user control over data and researchers’ need for data. The potential benefits of adopting a user-centered design approach to EULA development are reflected in the high proportion of clients that agreed to share data following completion of the process (421/478, 88/1%). This is noteworthy, given it has been argued that the length and complexity of EULAs have been used as an obfuscation strategy to increase the likelihood that people agree to terms that benefit those that receive materials [35,50]. However, our findings are consistent with previous research, suggesting clearer EULAs can lead to a greater number of consumers reading and understanding the terms, which can in turn increase the likelihood they accept them [51].

This study has significant strengths, including centering community partner perspectives, using a multiphase approach to incorporate participant feedback, and developing actionable steps to ensure ethical data sharing in eHealth technology. Limitations include the possibility of bias inherent to qualitative methods: facilitator age, social status, race, and participant involvement in the development of the EULA materials reviewed could bias their responses. Participants may have felt pressure to please facilitators (social desirability bias) and may have limited contributions due to discomfort (sensitivity bias). Another important limitation to note is the relatively small sample size, particularly in the client and family subgroups, which limited the ability to make subgroup comparisons. However, among the subgroups, the findings appeared broadly consistent, mitigating this as an issue. While there was high consistency at the participant level, indicating saturation, this may be partly attributable to group dynamics; data from additional focus groups would be informative, including from more diverse service users and their families with different language preferences and needs. Future work is already underway to include collaborating with partners who speak languages other than English to determine the best approaches for translating EULA materials in a culturally accessible and linguistically appropriate manner.

Limitations were minimized where possible: to lessen dominant respondent bias, facilitators promoted fewer vocal participants; to avoid reference bias, questions were ordered logically, minimizing swaying participants’ perspectives; to mitigate social desirability bias and sensitivity bias, facilitators positioned participants as the experts in their experiences and encouraged them to provide honest feedback and frame negative feedback as crucial to addressing potential issues; and to minimize reporting bias, we used codebooks, multiple coders, and participant feedback before finalizing themes. COVID-19 logistical barriers likely impacted provider recruitment among consumers. Relatedly, COVID-19 safety precautions necessitated videoconference meetings, excluding participants without adequate internet access or electronic devices and those uncomfortable with internet-based participation. Although cross-clinic videoconferencing likely increased the breadth of voices included in the discussion, this selection bias may be particularly relevant given the technology-oriented subject matter. Future research should examine eHealth technology and data sharing attitudes with individuals with low comfort with technology and who prefer in-person participation.

In a period of rapid expansion of eHealth technology availability, the contrast between community partners wishes for transparent, accessible data sharing agreements and the convention of EULAs being complex, convoluted, and centered on the needs of the developer presents a significant issue in the field. This study highlights the value of using community-informed research to identify community partners’ needs, values, and priorities around data sharing. Furthermore, when needs and values are incorporated into the EULA design process, this study demonstrates that the approach can lead to high rates of data sharing. This suggests that adopting a more ethical approach to data sharing can have the dual benefit of addressing community partner needs while simultaneously supporting researchers’ efforts to collect eHealth data.

## Appendix

Multimedia Appendix 1 COREQ checklist.

Multimedia Appendix 2 Part 1 focus group guide.

Multimedia Appendix 3 Descriptive ice-breaker questions for phase 1 focus groups.

Multimedia Appendix 4 Beehive end-user license agreement (EULA) video.

Multimedia Appendix 5 Part 2 focus group guide.

Multimedia Appendix 6 Questions presented to study participants to elicit feedback preliminary coding framework and supporting quotations.

Multimedia Appendix 7 Additional quotations supporting main themes.

## Abbreviations

COREQ: Consolidated Criteria for Reporting Qualitative Research
EP: early psychosis
EPI-CAL: California Early Psychosis Intervention Network
EULA: end-user license agreement
HIPAA: Health Insurance Portability and Accountability Act

## Data Availability

The data sets generated during and/or analyzed during this study are available from the corresponding author on reasonable request. Data will be available in the form of deidentified transcripts.
