# Supplementary material for: Incorporating Community Partner Perspectives on eHealth Technology Data Sharing Practices for the California Early Psychosis Intervention Network: Qualitative Focus Group Study With a User-Centered Design Approach
Source: JMIR Hum Factors. 2023 Nov 14;10:e44194. doi: 10.2196/44194 (PMC10685281; doi:10.2196/44194)
Supplement: Multimedia Appendix 2 [file humanfactors_v10i1e44194_app2.doc]

# Part 1 Focus Group Guide

# Introduction (10 minutes)

In December 2018, this service joined the Early Psychosis Learning Healthcare Network. As part of this network, additional data will be collected, immediately available to you, and used to actively support your treatment. This will provide clients and family members with an additional way to help identify and address their treatment priorities, and to track progress over time.

As we develop this platform, we want to keep in mind that the use of electronic personal health information is an important issue to patients, families, and providers. It is important that as patients, you have conversations about how these new innovations may improve the quality of healthcare. The purpose of this study is to understand your views on how your personal health information is and should be used. We will discuss sharing of sensitive information, informed consent of sharing such information, and your preferences for sharing different types of data.

Before we begin, we want to review some definitions as they pertain to a healthcare setting.

**Privacy:** Refers to the right of an individual to keep their health information out of public view.

- Example: You have the ability to control who can access your health record.

**Confidentiality:** An extension of privacy pertaining to keeping identifiable data private or secret. It refers to the duty of anyone entrusted with health information to keep that information private.

- Example: Information you share with your doctor should not be shared with anyone other than you and them (or a guardian) without your permission or consent.

**De-identified information:** Certain information associated with an individual is removed in order to protect their identity.

- Example: You fill out a health questionnaire for a research study which includes identifying information: your name and date of birth. Your name and date of birth are removed before this data is shared with outside researchers.

**Anonymous information:** Refers to information that has no direct or indirect identifiers at the time of collection and which cannot be linked to any individual.

- Example: You fill out a health questionnaire for a research study which does not ask for your name, date of birth, or any other information that could identify you.

# Part 1: Understanding of/Perspective on Data Sharing (RE: health information/treatment) (20 minutes)

*[zoom poll triggered prior to discussion](5 minutes)*

- What is your current understanding of how your mental health data is being shared?
  - What aspects are you aware of?
    - Does it affect the types of information you are willing to share?
      - How does this affect how you share?
- What is your current understanding of how your mental health data is being protected?
- What would make you more likely or willing to share your mental health data?
  - What can be done to make you feel more protected or ease your concerns?
- How might your preferences for data sharing differ between mental health and physical health?
  - How might your preferences for data sharing differ between health information and non-health information (ie. social media, apps)
- What are your thoughts about sharing identifiable data (name, DOB, address, etc.) knowing only you and your clinic team have access?

# Part 2: Changing sharing options (understanding idea of “living informed consent” or “the right to be deleted”) (20 minutes)

*[zoom poll triggered prior to discussion]*

- What is your understanding of informed consent?
  - What do you think “living informed consent” means?
- Under what situations would you feel the need to remove your health data from a digital technology application or health platform?
  - How important is it to have the option to delete your data?

# Part 3: Levels and type of sharing (aggregated, identifiable) (30 minutes)

*[Remind of definitions of identifiable vs. de-identified data]*

*[Present definitions of individual level data vs. group level data]*

- What type of information do you feel most comfortable sharing at the “individual” level (i.e., demographic characteristics, symptom levels, functioning)?
- What type of information do you feel most comfortable sharing at the “group level” (i.e., demographic characteristics, symptom levels, functioning)?

*[Present definitions of levels of data sharing: your clinician, your clinic team, other early psychosis clinics, study administrators, your county, the state, national data repository]*

- What are your thoughts about sharing your individual level health care data (disaggregated data) with other levels (clinicians at your clinic, study administrators, with the county, with a national data repository)?
- What are your thoughts about sharing your data at the grouped level (aggregated data) with other levels?

*[zoom poll triggered after discussion]*
