# Supplementary material for: Incorporating Community Partner Perspectives on eHealth Technology Data Sharing Practices for the California Early Psychosis Intervention Network: Qualitative Focus Group Study With a User-Centered Design Approach
Source: JMIR Hum Factors. 2023 Nov 14;10:e44194. doi: 10.2196/44194 (PMC10685281; doi:10.2196/44194)
Supplement: Multimedia Appendix 3 [file humanfactors_v10i1e44194_app3.docx]

Polling Questions for EULA Focus Group Pt 1

Pt 1: Understanding of/Perspective on Data Sharing *(asked after discussion of this section)*

1. **How comfortable are you with sharing your deidentified mental health data outside of your clinic for research purposes?**
   1. Very comfortable
   2. Somewhat Comfortable
   3. Somewhat Uncomfortable
   4. Very uncomfortable
2. **What would make you more comfortable with sharing your deidentified mental health data? (select all that apply)**
   1. Understanding the laws and regulations in place to protect my personal health information
   2. Knowing that my team is committed to protecting my information
   3. Having the option to change my mind about sharing my mental health data
   4. Knowing my mental health data will be used to try to improve treatment for others
3. ***(Providers only)* How comfortable do you think your clients are with sharing their deidentified mental health data outside of your clinic for research purposes?**
   1. Very comfortable
   2. Somewhat Comfortable
   3. Somewhat Uncomfortable
   4. Very uncomfortable
4. ***(Providers only)* What do you think would make your clients more comfortable with sharing their deidentified mental health data? (select all that apply)**
   1. Understanding the laws and regulations in place to protect my personal health information
   2. Knowing that my team is committed to protecting my information
   3. Having the option to change my mind about sharing my mental health data

Knowing my mental health data will be used to try to improve treatment for others

Pt 2: Changing shared options *(asked prior to discussion)*

1. **Have you ever changed your data permissions on an app / website that you use?**
   1. Yes
   2. No
   3. I didn’t know I could
2. **Have you ever requested to delete your data from an app/website you use?**
   1. Yes
   2. No
   3. I didn’t know I could

Pt 3: Levels and type of sharing *(asked after discussion)*

1. **What type of mental health data are you comfortable sharing outside of your clinic for research purposes? (check all that apply)**
   1. Individual level
   2. Aggregate Level
   3. Identifiable
   4. De-Identified
2. **Who are you comfortable sharing your de-identified, individual-level mental health data with? (check all that apply)**
3. Clinic providers/staff who will use it to guide treatment
4. County Officials who will use it to evaluate early psychosis clinics
5. California State Officials who may use it to change state laws or funding
6. Researchers (e.g., UC Davis Researchers) who will use it to improve treatment for others
7. National database where researchers around the world can use the data to understand early psychosis
8. ***(Providers only)* What type of mental health data do you think your clients are comfortable sharing outside of their clinic for research purposes? (check all that apply)**
9. Individual level
10. Aggregate Level
11. Identifiable
12. De-Identified
13. ***(Providers only)* Who do you think your clients are comfortable sharing their de-identified, individual-level mental health data with? (check all that apply)**
14. Clinic providers/staff who will use it to guide treatment
15. County Officials who will use it to evaluate early psychosis clinics
16. California State Officials who may use it to change state laws or funding
17. Researchers (e.g., UC Davis Researchers) who will use it to improve treatment for others
18. National database where researchers around the world can use the data to understand early psychosis
