# Supplementary material for: Incorporating Community Partner Perspectives on eHealth Technology Data Sharing Practices for the California Early Psychosis Intervention Network: Qualitative Focus Group Study With a User-Centered Design Approach
Source: JMIR Hum Factors. 2023 Nov 14;10:e44194. doi: 10.2196/44194 (PMC10685281; doi:10.2196/44194)
Supplement: Multimedia Appendix 5 [file humanfactors_v10i1e44194_app5.doc]

# Part 2 Focus Group Guide

# Introduction (15 minutes)

“In December 2018, your early psychosis clinic joined the Early Psychosis Learning Healthcare Network. As part of this network, your clinic will use an application called Beehive to administer surveys for clients, support persons, and providers. This survey data will be available to providers in a web dashboard and may be used to actively support your treatment. Beehive will provide clients and family members with an additional way to identify their treatment priorities, communicate priorities with providers, and track progress over time as part of regular care.

As we develop this platform, we want to keep in mind that the use of electronic personal health information is an important issue to patients, families, and providers. It is important that as clients, you have conversations about how this technology may improve the quality of healthcare. The purpose of this study is to understand your views on how the End User License Agreement in Beehive is presented.

When an individual first interacts with Beehive, they will watch a short video that briefly describes the application and the data-sharing options on it. We are going to watch that video together now and then we will ask for your feedback.”

# Part 1: EULA Video Presentation and Feedback (55-60 minutes)

*[Present Video (7 min)]*

- What information in this video did you already know? What was new?
  - What information wasn’t necessary?
- What kinds of questions come up for you when you watch this video?
  - If there was information you wish was in the video but wasn’t, what would you add?
    - When we talk about data sharing with NIH, what level of detail do you want to know? Everything about the relationship between NIH, ENDCC, Westat? Or just the general description that is provided?
    - When we give examples of how data will be used in research, we chose specific, concrete examples. Would a broader example such as “we will use this data to evaluate how Beehive impacts individual’s recovery” be preferred? Other ideas?
- How do you feel about specific vocabulary used in the application?
  - Application vs. Platform vs. App?
  - Early Psychosis Clinic team vs. Early Psychosis Team vs. Treatment Team?
  - National database vs. ENDCC?
- What do you think about showing a video to explain the EULA?
- Does this video make you more or less likely to want to use Beehive?
- What do you remember from the video?

# Part 2: Present EULA Flow in Beehive (15-20 minutes)

“Now we will look at the application flow after the video is complete.”

*[Present EULA flow in application, explain with following text]*

After watching the EULA video about Beehive, individuals will then review the full EULA text. In order to use the application, users must acknowledge that they understand three things: Beehive is not intended as a substitute for emergency services, their clinic and providers will have access to their identifiable data as part of regular care, and they can change their data sharing options at any time. Users may choose to opt-in to share data with UC-Davis and/or with NIH.

- What do you think about the descriptions used in this section?
  - Is it easy to understand?
  - Is it missing information?
- What do you think about the presentation/flow of this section?
  - Is it clear what is required vs. optional?

*[Rewatch the video for additional comments]*

# Part 3 (additional questions for clients/families): Control of Data/Minors (5-10 minutes):

- 1. How would you prefer to request to delete your data?
     1. Through clinic?
     2. Directly contact study?
  2. (Clients) What components are you most/least willing to share with NIH?

*(Researchers) Think about how we could allow clients to opt-in to share parts of data if they don't want to share all of data, ex. Share demographics but not symptom data)*

- 1. (Families/Clients) What should happen when a parent wants to share minor’s data but minor doesn't? Or vice versa?
