# Supplementary material for: Incorporating Community Partner Perspectives on eHealth Technology Data Sharing Practices for the California Early Psychosis Intervention Network: Qualitative Focus Group Study With a User-Centered Design Approach
Source: JMIR Hum Factors. 2023 Nov 14;10:e44194. doi: 10.2196/44194 (PMC10685281; doi:10.2196/44194)
Supplement: Multimedia Appendix 6 [file humanfactors_v10i1e44194_app6.docx]

Participant Feedback on Results

Q1 Did you review the themes and quotes from the group(s) you participated in?

- Yes
- No

Q2 Do you feel that these themes and quotes accurately represent the discussion that we had?

- Yes 🡪 Skip to Q4
- No
- Unsure/Can't Remember 🡪 Skip to Q4

Q3 Please explain how we have not accurately represented the discussion.

________________________________________________________________

________________________________________________________________

Q4 Please provide any additional comments you may have.

________________________________________________________________

________________________________________________________________

________________________________________________________________

Q5 Would you like to be notified of publications related to this research?

- Yes
- No 🡪 end survey

Q6 Please provide your email address to be notified of publications related to this research

________________________________________________________________
