# Supplementary material for: Incorporating Community Partner Perspectives on eHealth Technology Data Sharing Practices for the California Early Psychosis Intervention Network: Qualitative Focus Group Study With a User-Centered Design Approach
Source: JMIR Hum Factors. 2023 Nov 14;10:e44194. doi: 10.2196/44194 (PMC10685281; doi:10.2196/44194)
Supplement: Multimedia Appendix 7 [file humanfactors_v10i1e44194_app7.docx]

# Quotes from Phase 1 Groups

| Phase 1 Theme | Client Quotes | Support Person Quotes | EP Provider Quotes |
| --- | --- | --- | --- |
| **Discussion of Data Sharing of Health Data** | | | |
| Parity between physical and mental health data | I don't have any distinction. I'm very open about my mental health as well as my physical. (Client 3, GRP2) | Well, personally I don't feel that any of my medical history would need to be hidden, but if it was de-identified I wouldn't mind at all. (Parent 2, GRP3) | With regard to our clients, we always are in discussion about how important it is that physical and mental health are connected, and the idea that a team of professionals and support individuals, the more communication, the better… And when it comes to my own health information, I try to just, feel the same way. (Provider 10, GRP1) |
| Current Understanding of Data Sharing of Healthcare Data | I believe they call it HIPAA or– that protects a lot of your medical information, as well as a lot of your identifying information for mental illness and such. (Client 1, GRP2)  At the state level, they're the ones funding the different counties. By the group data that's sent to them, they know who to tax, how much they can tax them so that your clinic gets state funding, and your clinics are all in different counties. And whoever has the best productivity gets the most funding and the best interns and all that kind of stuff. (Client 4, GRP2)  My information is confidential, unless I sign for something to let that be disclosed. That even goes for close family members. (Client 3, GRP 2) | Anytime data needs to be shared, I have to sign a paper to give permission. (Parent 1, GRP3)  I haven't really thought about it. **I** do feel like it's safe, but I'm not sure what I have to support that. (Parent GRP3) | I know that health insurance …companies can access it. … They use that for reimbursement purposes…. So, I think there's multiple other outside companies and organizations that can probably legally have access to it that and maybe the patients are not even aware of… I think they have access to notes, for example, and diagnoses. (Provider 5, GRP1)  Research side might be behind locked cabinets or something like that with a random number assigned to the file. (Provider 3, GRP1) |
| Preferences for Sharing Healthcare Data: Previous Experiences |  | And I will say my son got dinged by the DMV, due to hospital stay. why would you do that if his record is way cleaner than mine driving-wise? There was no reason for him to get that letter in the mail saying you're going to be suspended if you don't show up at this court hearing. And that's how it was derived from the hospital stay. (Parent 3, GRP3) | I think my positive and negative biases are related to the fact that I've worked in clinical research for 20 years. And I wrote somewhat comfortable on both answers, because I know at our clinic, we're super careful about how we collect**.** (Provider 2, GRP1)  I've been in a lot of situations where …parents get reunified with kids and they end up in the [EP program] or in our regular behavior health. And parents don't want any of the past information from CPS reports or anything that's come up in the past that may also be happening in treatment that may inform treatment. (Provider 6, GRP1) |
| Preferences for Sharing Healthcare Data: Purpose or Reason for Health Data in General | Right now it's presented where you have to sign this release or I'm not going to go through with this session. I can't give you any services because that goes against your insurance. (Client 3, GRP2)  If my therapist was going on a vacation leave, and then a new therapist was taking over, I think some basic information I'd at least want them to know, is my name, my age, I'm working or going to school, who I live with. If I hang out with friends, what my formal diagnosis is. I think these are all important things. (Client 4, GRP2)  … I'm more comfortable sharing my information knowing that it's going towards helping other people. And also funding too because I know that's definitely important with further helping others as well. (Client 2, GRP2) | …if I put my data in here and we do come up with a solution that actually would help my particular client, will that funnel back down to me in order to get the help so I can get the help results also back out of it? (Parent 3, GRP3)  I think if all that data is shared, hopefully there'll be more programs or there's a lot, programs to keep the patients longer or more outpatient treatment that is available. (Parent 2, GRP3) | I feel like our clients would ask, "What is going to be shared and why? What's the benefit of doing this?" (Provider 2, GRP1)  If it's required information in order to make sure that my health insurance pays for my services, right? Then it's a level I have to be okay with it in order to be able to receive the services**.** (Provider 6, GRP1)  So I think we definitely try to be really thorough about how we're going to use their information but also ensure that they are going to benefit with also the assessments and the treatments that we do. We give them summary reports of our findings. So that has been really valuable to folks**.** (Provider 7, GRP1)  … I think most clients would love to hear that anything we do is going to change policy. That's one of the reasons they often join our program is they actually see us implementing the research and the data that we get from the research we do. (Provider 2, GRP1) |
| Preferences for Sharing Healthcare Data: Identifiable Data | I think having all that information out there could be really dangerous especially for people want to use it for different purposes or commercial purposes especially. But for people like I can personally meet or clinics and stuff, or maybe even researchers, maybe that stuff, I'm a little more comfortable with. (Client 3, GRP2)  I'd be less comfortable with the closest to me like the provider. Then they really get to see all of you and they can judge you and evaluate you, come to a conclusion. That's more tailored to who you are as a person than someone at a clinical or state level. Wherever you go you just get more and more of a statistic. I don't know, I have mixed feelings. (Client 1, GRP2)  Whenever I'm applying for a new job, I'm very cautious of how they will look at my social media profiles so that I'd either omit some information but also highlight other information that I want them to see. (Client 4, GRP2) | If people knew or know that their health data could be used against them, they would be up in arms... But if health data could be used where we could have special accommodations, “wow, oh, sure, I'll take that”. And we don't know if this is detrimental to our way of living or how people treat us. (Parent 4, GRP3)  Well, my son who is 18 now, he started [a social media platform] when he was 15…. And a lot of the comments he made were very immature and I thought, well, if you're trying to get a job and people see that, that's not good. (Parent 2, GRP3) | [At the] state level I think more aggregate would be more appropriate. …But I do think it almost gets more and more sensitive as we go versus more trusting. (Provider 3, GRP1)  When I was younger, I went through something that was very challenging health wise. And I did not feel very comfortable with the rest of my providers knowing anything about it. And so I specifically asked that it was not shared. And I denied doing ROIs between that doctor and other people for actually quite a bit of time until I was older and I felt a little better**.** (Provider 6, GRP1)  A lot of my clients …[are] concerned about what they reveal, what consequences they might bring in terms of… employment. If someone disclose something they had a background check, would it hinder their chances of getting a job? (Provider 1, GRP1) |
| Preferences for Sharing Healthcare Data: De-identified data | I think [sharing data at an aggregate-level] would open up my feelings about what I'm comfortable with sharing, so I can share everything. (Client 1, GRP1) | Again, I think if you're not being identified I'm always willing to share a little bit more as we're not going to be individualized. (Parent 3, GRP3) | It's such a zoomed out look, where you don't feel like you could be personally held responsible for the way that you are and you won't really feel stigma (Provider 6, GRP1) |
| **Discussion of Data Sharing of Non-Health Data & Sharing Data with Commercial Entities** | | | |
| Understanding of risks | Once you put something out there, it is incredibly hard to get it back. So, especially with all these trackers and things watching you and advertisements, it becomes like a sinking ship with a tiny bucket. You could delete a tiny little bit of data but in the long run, I feel like it's not going to do much. (Client 3, GRP2) | People can just Google you and find out all this very personal information about you. Not everyone has the best intentions. (Parent 1, GRP3)  [A big technology company] just bought a large data bank of health information of, I don't know how many, 15 million people, and they claim it's all for research. If I'm in there, I didn't give that permission for them to have that. So somebody sold out. (Parent 4, GRP3)  Just within this conversation, I was thinking we're really secure. And then all of a sudden, boom, I'm not so secured anymore if I know 10 years down the line somebody can take that information and twist it. (Parent 3, GRP3) | I feel like no data is safe. Once you release it onto the internet especially because of all the articles saying that there was a breach with this site and they have your credit card information. (Provider 1, GRP1) |
| Understanding of protections | I think there's still some websites where they make it really accessible to be able to obstruct your data, like you can make your account private. You can alter an entry, you can scrub whatever metadata is on there. There are options. (Client 4, GRP2) | This happened to [a social media platform] where [it] actually has to reveal and allow you to download every post that you have ever made before you say deactivate. But not every website has that, but [this social media company] trying to build credibility did that. (Parent 4, GRP3) |  |
| **Actionable Preferences for Researchers** | | | |
| Transparency | I think transparency is pretty great. Being able to put in a request for information, see what you get sounds like a really useful tool. I think something that'll also help would be having a list or some kind of a form to show you Who's getting what information**.** (Client 3, GRP2)  When you start mixing mental and personal health information with commercial benefits, things can get sketchy. Because I feel if that happens, it's going to start building incentives for providers to get more and more information out of people.(Client 3, GRP2) | I just feel like I should be able to know who's accessing what, when, and why. You know? (Parent 1, GRP3)  Kinda like our credit report. We know if it has been accessed (viewed) (Parent 4 written comment, GRP3)  …my son has done a lot of research and things like that. So I'm not sure how the data is used, and it would be nice to be able to see how it's used and to make comparisons in our own situations also. (Parent 3, GRP3) | I teach all our trainees to learn the consent form forward and backward, but also know how to talk about it in layman's terms, so that people really understand and make sure they understand what they're saying...I've had patients that are extremely symptomatic, but they'll say, "[Provider], I wasn't going to fill it out, but the way you explained it, I actually think I feel comfortable now." … (Provider 2, GRP1)  I think culturally as well. I saw a difference between my traditional American versus an immigrant family, right? So I knew that I had to spend more time explaining or highlighting on some of the important pieces it’s sort of strategic as well just understanding someone's history. (Provider 3, GRP1) |
| Data Protections | I would say that sometimes my diagnosis between very curious situations where I might be on some drugs, that totally tranquilize my baseline. And I might be more susceptible to just sign whatever you put in front of me. Because I'm not fully confident at that moment. I'm vulnerable and, I just understand that this is all just a formality to just continue receiving the services that I absolutely need in that moment to recover. (Client 4, GRP2)  I think that many times when these documents are signed or they are disclosing how I'm going to be releasing your information, that oftentimes it’s just me and the provider in the room. I don't have a social worker and a family member or a friend or even a peer support specialist. And that's a very intimidating situation. I feel alone in that moment where I can't look left and right and get a head nod of, yeah, this is okay to do. (Client 4, GRP2) | But I need to know what is the formula [to de-identify data] like. You've described it to me, but that doesn't give me the confidence to really give you a thumbs up. (Parent 4, GRP3) | We have to be prepared to talk about that because clients say to me, "Wait, there's a what? You have to do what to protect me? I didn't even know this was." … I think the more transparent we are, and the more we educate, that's always better because people need to know well, this is what's going on, these things are out here to protect us, all of us. (Provider 2, GRP1) |
| Control Over Data | There's so many protections on my information that even I can't access it, which I find really ridiculous. … Why would I want you to share that information to other people if you won't even share it to me? (Client 4, GRP2)  I think [the ability to delete your data] is a fairly important option. If at the very least for the peace of mind it can give. (Client 3, GRP2) | I will give you what you need so that I can receive the service. You can render your services, but I want to give you the minimum data. (Parent 4, GRP 3)  It's like a no regrets kind of thing. If I change my mind later I can just be like, well, I felt differently about it. I thought about it and I don't want to do that. (Parent 1, GRP3)  Different sharing levels at different processes where people are in or what stages they are in, may require different data collection ways…And it's not a security level they're worried about, it's the comfortability level of just their own contemplating of what's going on and processing everything. (Parent 3, GRP3)  I think it provides you with a level of security. You feel secure if you have the ability to do that. And if you feel secure you put more information almost out there. (Parent 3, GRP3) | We have clients that are minors, right? So say for example, the way that they identify [their gender] is something that they're not out to their parents or their family, but they want to be used. At the end of the day, their parent has to sign whatever document that they just filled out. …. I've always liked having two different mental health sheets that we have one the youth only fills out and then the parent gets to fill out the same information but what they think for the kid. (Provider 6, GRP1)  I think people just feel a little more comfortable that they know they can change their mind. (Provider 2, GRP1)  I would feel a lot of comfort in knowing that I had the option to delete all of my data from a system so that no one can go back and find that I had received services from that particular provider. (Provider 9, GRP1)  I think it would be great for all PHI, Protected Health Information to have the option for that to be deleted, I think is something, a right that we should all have. But when it comes to de-identified data, at some point, it's too late. (Provider 10, GRP1) |
| Rapport |  |  | I think rapport with our patients is really important…. I think there was something about the rapport building up front from the phone line to actually consenting that was much more comfortable compared to just someone new coming in and explaining the consent that they had never had contact with or any relationship with prior. (Provider 3, GRP1)  I noticed my health care provider, they ask me if I can share my information to be part of research, but they didn't explain what kind of research so I could know. So, that was something that I was thinking about thinking about medical because I would want to know what is my health information, what kind of research they're doing. (Provider 7,  GRP1) |

# Quotes from Phase 2 Workshops

| Phase 2 Theme | Client Quotes | Support Person Quotes | EP Provider Quotes |
| --- | --- | --- | --- |
| **Implementation of Actionable Items** |  |  |  |
| Control over data | I also like how they presented the option where you could stop or delete the information you provided. I think it also did a great job of explaining how if you opt out or if you chose to present your data to the NIH, that if it has already been used, you can't delete that part. I thought that was really nice as well. I like the ending emphasis on your, how it says ‘it's your data you're in control’- I think that's really a nice way to end off the video. (Client 1, GRP5) | And I liked the data opt. Just for maybe six months, I don't want to participate in this and that makes me feel, or my treatment go along better. I think those are the most important things of the app and the whole running of it. I think that that gave them a lot of self-control over it. They feel like they don't have a lot of self-control over things or even their life and this gives them control over at least this portion of it. And asking the questions beforehand to get permission before you put in any data, I think is an awesome idea, just on an interim level. (Parent 2, GRP6) |  |
| Protections | It's like a big research opportunity and very uninvasive. Because it has data that protects your identity, but still allows it to be used by researchers to treat people like me. (Client 2, GRP5) | The explanation and the data that was limited to them, as far as the percentage of just how many people are on medication per zip code or whatnot, that's definitely pertinent. (Parent 2, GRP6) | [The message I came away with was] That my health information would be protected. (Provider 4, GRP4) |
| Familiarity/Rapport | I do agree that agreeing to UC Davis would be like agreeing it to you guys because we've met you guys. But the people at NIH I don't think I'd ever meet them in person, so it would be like sharing it with a stranger. (Client 3, GRP5) | I think you have a relationship that you build with your clinician and the support people that you have immediately around you, and those are your go-to people for most things. (Parent 2, GRP6) | Is there a way to possibly present this later on after they've gotten to know the team and feel more comfortable of what the program is about and get used to people? (Provider 11, GRP4) |
| Familiarity/Reputation | I'd be curious what other research Westat has done before… if they have some sort of reputation with other projects they've done, then I'd be much more trusting. (Client 3, GRP5) |  |  |
| Transparency/Present in Materials | It's important how it lets you know that if any information has already been shared with other researchers that it will not be deleted. For people who may feel indifferent or a little on the fence on if they want to share that data, that might help them to decide if they're going to leave that box unchecked or not. (Client 1, GRP5) | I agree with [Participant 2] and [Participant 1]. I like how it made it very clear, like how your data is being shared and how you can opt out and also making it clear that the information that has already been shared is just there. (Parent 3, GRP6)  I think with the concern with who the data gets shared with in the beginning and at the end, the summary there with the understanding of my data will be used for this, this, this, and you have the power to delete it, I think opening and closing with that is definitely effective and the questions are easy to read. (Parent 2, GRP6) |  |
| Transparency/Need more Information | I was wondering, can you choose to only have one kind of data submitted or both have to necessarily be intertwined and put together? Because they didn't make it very clear in the video whether you could choose to have either or. It just seemed like both were automatically together. (Client 2, GRP5)  I think the term deletion feels a little bit misleading. Because if my data is already put in a database and it's been downloaded, then it can't really truly be deleted. (Client 3, GRP5)  I guess like examples of how information will lead to better treatment. It's what kind of impacts does it have? Does that mean there are going to be more therapists in my clinic, or that there are going to be more clinics like that in my area so it's more accessible to me? Does the increased research allow more participants to find clinics like the ones I’m at? (Client 3, GRP5) |  | …maybe it might be helpful to have a section of like dismantling some myths about what it means to participate in this bigger way like this. We are not doing sharing this information with their local government or some way of really, disclaimer of what we're not doing, that's more obvious might be helpful. (Provider 3, GRP4)  So, I wonder if we could say, because this isn't part of the benefit, we're going to be at a track from session to session and see how they're doing, maybe emphasize that like that. Their clinician will be able to use it. (Provider 14, GRP4)  Going back to the diagram of how do we make this more of a visual for those who are having a hard time tracking something like this on almost using a diagram on a micro level. How does this directly help me and then more on a state level and it keeps growing there? (Provider 3, GRP4) |
| **Presentation Method** |  |  |  |
| Impact on Transparency | I guess in the past with EULAs it's like they're already pre-typed in to the form. If I didn’t want to have it sent to somewhere, I would physically have to cross it out. But since you guys have checkboxes that's much more convenient. (Client 3, GRP5)  I personally think it's important to separate them. Letting you know that it's been shared with UC Davis and also with the NIH. (Client 1, GRP5)  I think it just depends. If the video comes on and then it's the EULA right after it and I'm scrolling through it, and if I just press two of them and then press confirm, and it knocks me with a notification saying, "You have to check all three boxes." Then I'm going to look at my therapist and be like, "Hey, what's up?" (Client 3, GRP5) | … I agree with that, with both [Parent 1 and Parent 2], just making it clear what's optional and what's required. (Parent 3, GRP5) | When I'm going through an intake packet with a client I'll typically I don't like leaving forms blank … I don't want to put a blank form in their chart. I actually have them write decline if they are going to decline it. Would it be possible to have a decline function in there if they choose to opt out of NIH and opt-in to UC Davis or if they choose to opt out of even the clinic using Beehive? (Provider 12, GRP4) |
| Impact on Clarity | Something that I didn't know was the de-identified data. When they were describing that I thought it was well described because I didn't know what it was before. (Client 1, GRP5) | Yeah, but yeah, no, it created a nice, clear picture. I know when we first met, I didn't know anything about the program and it took me a while to kind of digest what we were talking about and share feedback. (Parent 1, GRP6) | I think I see what you guys are trying to do by keeping it animated and interesting for people, to visually capture the eyes… But for me, I was just, it was too much for my eyes while trying to also listen to all this information that was being given. (Provider 4, GRP4)  I think some part of the artwork helps. It's another tool to rehearse and just to have an image. But I don't know, but the robot, for example, is time consuming. It's pretty cool but I don't know whether that actually added something, where the safe, I think it actually makes sense, because it's the visual that your data are safe. (Provider 4, GRP4)  As we're putting in those pauses, if there could just be a chunking of the information that was just provided, that's going to be a process that we're going to repeat in our treatment as well. (Provider 12, GRP4)  I'm not sure whether it's part of the legal obligation to use the terms like EULA or when you explained the limited data set… But I'm wondering if you actually need to use that term because I wonder if that's just more information that people have to hold in their mind that maybe isn't as necessary. (Provider 14, GRP4) |
| Impact on Engagement | I've always been the type of patient that wants my information to help others. I know that this application is a heavy emphasis on using the data and research to use for or to help assist with that type of stuff in the long run. (Client 1, GRP5)  I think an interactive experience would help you provide more learning from just watching a video. Because when you have a tutorial like in a video game, you have to click each link and you are given examples and you figure out how to do it. In a video … if you don't understand something and you have a question about something, it just goes past, you don't really get a chance to elaborate on what you think.  (Client 2, GRP5) | I feel like just knowing my daughter's personality, that if it was the same questions that were at the clinic, that were also going to be on the app, that she would probably be open to doing it. (Parent 3, GRP 6)  …[My son] has delusions so… he doesn't want to write it down because he doesn't want people to know. He's just paranoid, so whether he answers it for the doctor, if it's on paper, but usually he doesn't want to record anything. (Parent 1, GRP6) | For my more reserved patients, they might decide to just do the initial my clinic, and that's it because of the lack of information or ability to process what these other two means. I don't lose an opportunity there of having them sign up in this bigger way and contributing in this bigger way, just because they felt compelled to just answer quickly. (Provider 3, GRP4)  We're working with clients who have early psychosis. A lot of them have suspiciousness, and paranoia and not trusting of people to present this at the start of their treatment might be off putting. (Provider 11, GRP4) |
